# Acknowledgement to Reviewers of *Toxins* in 2018

**DOI:** 10.3390/toxins11010028

**Published:** 2019-01-09

**Authors:** Toxins Editorial Office

**Affiliations:** MDPI, St. Alban-Anlage 66, 4052 Basel, Switzerland

Rigorous peer-review is the corner-stone of high-quality academic publishing. The editorial team greatly appreciates the reviewers who contributed their knowledge and expertise to the journal’s editorial process over the past 12 months. In 2018, a total of 515 papers were published in the journal, with a median time to first decision of 14 days and a median time to publication of 35 days. Among the reviewers who reviewed for *Toxins* in 2018, the following two have been selected to receive the “*Toxins* 2018 Outstanding Reviewer Award” for the timeliness and quality of their review reports in 2018:

Tolosa, Josefa from University of Valencia, Spain

Yoshinari, Tomoya from National Institute of Health Sciences, Japan

The editors would like to express their sincere gratitude to the following reviewers for their cooperation and dedication in 2018:
A. Manderville, RichardHsieh, Hsi-LungPoniedziałek, BarbaraAbass, KhaledHu, ChenlinPoór, MiklósAbd El Hakim, YasminaHuang, I-HsiuPortaro, FernandaAbraham, AnnHuang, Ren-YeongPosthaus, HorstAbrunhosa, LuisHuang, YadongPovey, AndrewAccinelli, CesareHübner, FlorianPredel, ReinhardAccoroni, StefanoHungerford, JamesPreece, Ellen P.Adams, VickiHwang, Soon-JinPrentis, PeterAdhikari, RajanIatsenko, IgorPrescott, John F.Agarwal, TinaIglesias, Maria Del RosarioPrevost, GillesAgha, RamsyIijima, NoriakiPrice, Joseph A.Agriopoulou, SofiaIkarashi, YoshiakiPriel, AviAinsworth, StuartIkeda, YasumasaProft, ThomasAird, StevenIkonomopoulou, Maria PPrzybilski, RitaAjandouz, ElhassanInaba, HiroshiPrzybylska-Gornowicz, BarbaraAkio, NakaneInagaki, HidetoshiPucca, ManuelaAlbrecht, Eric A.Inui, HideyukiPuddick, JonathanAlder, JaneIppoliti, RodolfoPunga, Anna RostedtAlfinito, EleonoraIrakli, MariaQuinton, LoïcAlfonso, AmparoIvanova, BojidarkaRadcliff, Fiona JaneAli, NurshadJabbari, BahmanRadmila, PavlovicAlmeida, C. Marisa R.Jackson Woo, Chit ShingRaine, RobinAloisi, Anna MariaJackson, LaurenRamachandran, AnupAlonso, EvaJackson, Timothy N. W.Ramalho, TeodoricoAlshannaq, AhmadJamwal, RohitashRasmussen, Peter HaveAlter, KatharineJin, ZhaoRaunser, StefanAlthaus, MikeJohnson, Stephen R.Raut, SangramAmasheh, SalahJones, MartinaRediske, Richard R.Amato, JussaraJosenhans, ChristineReed, KentAmes, Cheryl LewisJosic, DjuroReed, Kevin F.M.Ameye, MaartenJuan, CristinaReguera, BeatrizAnadon, ArturoJuan-García, AnaReinke, AaronAnadón, ArturoJuge, NathalieResiere, DaborAnas, Andrea RoxanneJunqueira-de-Azevedo, Inácio L.M.Restivo, DomenicoAnderson, GaryKahlert, StefanRestivo, Francesco MariaAndjelkovic, MirjanaKaito, ChikaraReveillon, DamienAndrade, FernandoKalb, Suzanne R.Reverberi, MassimoAndrade, María J.Kameneva, PolinaRiccardo, Zenezini ChiozziAndreev, KonstantinKamitani, ShigekiRicelli, AlessandraAnniballi, FabrizioKamm, ChristophRichardson, SimonAppell, MichaelKammerer, RichardRiehle, MichaelAranda-Rodriguez, RocioKang, TaejoonRighetti, LauraAraujo, PedroKaplan, ShaunaRitieni, AlbertoArgiles, AngelKarlovsky, PetrRizzo, CarmenArino, AgustinKarp, Barbara IllowskyRobin, CharlesArmstrong, GlenKashiwagi, ShinichiroRobinson, Samuel D.Arola, Henri O.Katikou, PanagiotaRodrigues, PaulaAterini, StefanoKato, KeitaRodriguez De La Vega, RicardoAung, Meiji SoeKawashima, HidekiRodríguez, AliciaAuvynet, ConstanceKędzierska, BarbaraRodríguez, Armando AlexeiAwad, MilenaKellenberger, StephanRodriguez, FranciscoAzarnia Tehran, DomenicoKeller, JuliaRodríguez-Carrasco, YelkoBacchiocchi, SimoneKhezri, AbdolrahmanRoelen, BernardBae, TaeokKikuta, ShingoRojo, Maria AngelesBailly, Jean-DenisKim, KyounghyunRomero, AlejandroBajpai, RichaKim, Seong-GonRos, UrisBakke, MereteKim, Tae KonRosier, Peter F. W.Ball, JonathanKim, Young-KwanRossi, FilippoBanerjee, DipakKimura, HidetoRothkoetter, Hermann-josefBang, Moon SukKimura, TadashiRoué, MélanieBarbier, MarietteKiris, ErkanRourke, WadeBarbirz, StefanieKirst, HenningRoze, Ludmila V.Barreira, João C.M.Kizis, DimostenisRuiu, LucaBarrientos Velazquez, Ana L.Klähn, StephanRuiz-Gómez, GloriaBartolotti, MassimoKleinteich, JuliaRyu, Ji-wonBasak, Ajit K.Klotz, JamesRzymski, PiotrBasti, LeilaKluess, JeannetteSabatier, Jean-MarcBattacone, GianniKlüß, JeannetteSachkova, MariaBaxter, SimonKoch, AlexanderSainz, Maria J.Beaulieu, DeniseKoch, MatthiasSaito, HideyukiBenedik, MichaelKoelman, J. H. T. M.Sakaguchi, YusukeBerghoff, BorkKoh, Cho YeowSala, AmbreBernardos, AndreaKohli, Gurjeet S.Salmaso, NicoBernhoft, AkselKomori, YumikoSalud Bea, Roberto De LaBerrada, HoudaKonno, KatsuhiroSandle, TimBerruyer, RomainKoo, SunwooSanny, Charles G.Berry, JohnKool, JSantibanez-Lopez, Carlos E.Bertuzzi, TerenzioKoppe, LaetitiaSantini, AntonelloBiałoń, MariettaKopra, KariŠarkanj, BojanBianchi, Daniela ManilaKosters, AstridSarrocco, SabrinaBideshi, DennisKouadio, James HalbinSatake, MasayukiBieczynski, FlaviaKovacs, MelindaSato, EmikoBilliald, PhilippeKovbasnjuk, OlgaSato, ShigeruBilska, KatarzynaKrähenbühl, StephanSatoh, HisashiBinz, ThomasKrieter, Detlef H.Saviola, AnthonyBlackwell, BarbaraKrock, BerndSavoie, Jean-MichelBlanco, Juan C.Krstanović, VinkoSawa, TeijiBlasi, JuanKu, SeockmoSberveglieri, VeronicaBocian, AleksandraKubo, TaiSchaumburg, FriederBöhm, JosefKuno, ToshiyaSchembri, MarkBoomer, Jonathan S.Kuo, Cheng-ChinScherer, Pia I.Bordes, PatriciaKuraku, ShigehiroSchneider, BarbaraBosilevac, JosephKwatra, Shawn G.Schroeder, ChristinaBotana, Ana MaríaLadeira, CarinaSchrum, AdamBotti, MaddalenaLagana, AldoSchwan, WilliamBouaïcha, NoureddineLam, Kwok HoSchwartz, HeidiBourne, Christina R.Larabee, Jason L.Schwartz-Zimmermann, Heidi ElisabethBovee, Toine F.H.Larsson, Michaela ESchwerdt, GeraldBowden, BruceLatorre, Juan DavidSebastian, AimyBradley, Walter G.Lattanzio, Veronica Maria TeresaSechet, VeroniqueBreidbach, AndreasLauritano, ChiaraŠegvić Klarić, MajaBrewer, MichaelLaustsen, Andreas HougaardSeifert, Steven A.Brigotti, MaurizioLe Hégarat, LudovicSemeraro, FabrizioBrinchmann, Monica FengsrudLeão, Pedro N.Seo, Jung-KilBroadwater, MaggieLebar, MatthewSeok, HyunBrown, Kyle E.Lebrun, BrunoSeubert, Erica L.Brvar, MiranLee, Hye SukSharkey, LiamBryła, MarcinLee, Kuo-KauSharma, BheshBrzozowska, EwaLee, Moo-SeungSheikh, AlaullahBuchanan, RobertLee, Yeon-JuShen, ZhenyuBukaveckas, PaulLehman, Peggy W.Shiomi, KazuoBurstyn-cohen, TalLemichez, EmanuelShityakov, SergeyBurtey, StephaneLePendu, JacquesShoemaker, CharlesBurtey, StéphaneLeung, Yuk-manShukla, Sanjay K.Busse, LilianLevesque, CelineSidiropoulos, ChristosButtenschoen, KlausLewin, MattSiebers, RobCadel-Six, SabrinaLewkowski, JaroslawSilva, AnjanaCaetano, MarianaLi, JihongSilvestri, IdaCai, ShuoweiLi, PengchengSimm, RogerCajado-Carvalho, DanielaLi, ShitaoSimon, StephanieCalvete, Juan J.Li, ShiyouSingh, Bal RamCâmara, José SousaLicea Navaro, Alexei FedorovishSingh, HarinderCampbell, CoreyLin, Hsu YangSingh, Kumar SaurabhCastella, GemmaLing, ChenSingh, SarbjitCatanzaro, ElenaLiu, QianqianSingharoy, AbhishekCerasino, LeonardoLlewellyn, Lyndon E.Sionov, EdwardCeruti, StefaniaLlirós, MarcSmith, DavidChalivendra, Subbaiah C.Lomonte, BrunoSmith, G. JasonChalyan, TatevikLopes Fuly, AndréSmith, JenniferChan, King MingLópez, Miguel ÁngelSmout, MichaelChan, LeeLopez-Moya, FedericoSolcan, CarmenChanda, AnindyaLorè, Nicola IvanSoler Aznar, MariaChang, Long-SenLorenz, NicoleSolfrizzo, MicheleChang, MinsunLoris, RemisSondergaard, Teis EsbenCharvat, Robert A.Louzao Ojeda, M. CarmenSondgeroth, KerryChassagnon, IreneLowe, RohanSong, ImsookChatterjee, JhinukLuo, MengSong, JeongminChen, ChenLuo, WentaiSørensen, Jens LauridsChen, Jack J.Ma, XiaochaoSorrenti, ValeriaChen, LongMa, YongSoulage, ChristopheChen, TianbaoMacKenzie, LincolnSousa, SandraCheng, Luisa W.Macori, GuerrinoSoutourina, OlgaChijiwa, TakahitoMagalhães, Geraldo S.Sparks, DarrellChilaka, Cynthia AdakuMahapatra, Ajit K.Spiliopoulou, IrisChirumbolo, SalvatoreMajumdar, RajtilakSpyros, GkelisCho, YukoMalek, NaveedSrivastava, SaurabhChoudhari, ShyamalMammola, Caterina LoredanaSrivenugopal, KalkunteChurro, CatarinaMancheño, JoséStaal, JensChyau, Charng-CherngMancianti, FrancescaStadler, MarcCianchetti, CarloManda, GinaStanford, KimCiriaco, FulvioManes, JordiStatsyuk, Natalia V.Citores, LuciaManfrin, C.Stearns-Kurosawa, Deborah J.Clemetson, KJManganelli, MauraStella, SimoneCohen, GeraldMangum, JonathanStępień, ŁukaszCollett, Mark G.Manjunath, ManuboluSterling, TracyComte, KatiaMantle, PeterStewart, George C.Confer, Anthony W.Mantzoukas, SpiridonStiles, BradleyConte, AntonellaManville, RíanStoev, StoychoConti, StefaniaManyes, LaraStorer, Nicholas P.Cook, DanielMaragos, ChrisStrickland, JasonCortinovis, CristinaMaresca, MarcSubbiah, MuruganCosta, João G.Margres, Mark JSugita-Konishi, YoshikoCosta, PedroMarie, BenjaminSulyok, MichaelCostello, Ben De LacyMarin, José Juan GarcíaSun, ChangpoCotella, DiegoMarin, SoniaSunagar, KartikCoutte, LoicMarinelli, LucioSundheim, LeifCowardin, CarrieMarino, AngelaSung, Wang ChouCreamer, RebeccaMarion, Jason W.Surup, FrankCruz, CeliaMariscal, VicenteSusca, AntoniaCuccioloni, MassimilianoMarla, SandeepSuzuki, TakafumiCumming, JonathanMarocco, AdrianoSuzuki, ToshiyukiCunha, SaraMarte, AntonioSwain, MartinCzuprynski, CharlesMartinez-del-Pozo, AlvaroSwan, Sarah C.Da Silva, AnaMartins, Mariana RenovatoSwevers, LucD'Agostino, PaulMaruyama, KeiSycha, ThomasDall'Asta, ChiaraMasago, YoshifumiSykłowska-Baranek, KatarzynaDaly, NorelleMascher-Frutschi, FabioSzabó, AndrásDänicke, SvenMasuda, HisakoTabashnik, BruceDaniels-Wells, Tracy R.Masuyer, GeoffreyTadahiro, SuzukiDavid, MichaelMatsumoto, TakuyaTannous, JoannaDavid, Michael Z.Mattei, CesarTartaglione, LucianaDavletov, BazbekMatthias F., MelzigTashima, Alexandre KDe Girolamo, AnnalisaMayer, ElisabethTashima, ToshihikoDe Haro, LucMaynard, JenniferTatters, Avery O.De Marcos Ruiz, SusanaMazor, OhadTatulian, Suren A.De Middeleer, GilkeMcClain, MarkTaylor, Erika A.De Pauw, JokeMcClane, BruceTaylor, MatthewDe Santis, BarbaraMcCormick, JohnTellez, GuillermoDeb, SubrataMcgrath, SaraTerzi, ValeriaDegen, GiselaMcKeague, MaureenTester, PatriciaDegola, FrancescaMcLaughlin, John E.Teter, KenDéleris, PaulMcNutt, PatrickTetreau, GuillaumeDellafiora, LucaMebs, DietrichTharad, SudaratDerman, YagmurMeca, GiuseppeThomas, Y. K. ChanDessain, ScottMegighian, AramThyagarajan, BaskaranDi Nunzio, MattiaMehbub, Mohammad FerdousThyparambil, AbyDi Stefano, VitaMeijer, HJGTien, Lu-TaiDiaz, DuarteMeijers, BjörnTietze, AlesiaDidier, AndreaMercader, JosepTisi, RenataDolgova, OlgaMercier-Bonin, MurielTocmo, RestitutoDong, MinMerda, DéborahTolosa, JosefaDossena, SilviaMetcalf, JamesTombelli, SaraDoxey, AndrewMeulenberg, ElineTomczyk, ŁukaszDoyle, ThomasMeyer, Kevin A.Toporowska, MagdalenaDucancel, FrédéricMeyer, Timothy W.Torres, JaumeDucatelle, RichardMézes, MiklósTorres-Russotto, DiegoDupieux, CélineMichlmayr, HerbertTorroba, TomásDurán-Riveroll, LorenaMiedaner, ThomasTortorella, DomenicoDurek, ThomasMikheyev, Alexander S.Toyotome, TakahitoDuringer, Jennifer M.Miles, JohnTrachtman, HowardDziga, DariuszMiller, DavidTreu, RolandEdwards, Adrianne N.Minervini, FiorenzaTrim, CarolEdwards, ChristineMishra, Rupesh K.Trim, Steven A.Eibl, Martha M.Mitchell, JohnTripathi, AshootoshEkateriniadou, LoukiaMitulovic, GoranTrojanowicz, BoguszEl Khalloufi, FatimaMiyashita, MasahiroTubaro, AureliaEl-Seedi, Hesham R.Miyoshi, Shin-ichiTufféry, PierreEspinosa, ManuelMoar, William J.Turrini, EleonoraEtxebeste, OierModahl, CassandraTzika, EleniFakhoury, AhmadModica, Maria VittoriaUmemura, MycoFalconer, IanMohammadi, ShabnamUpadhyaya, InduFalconer, Ian R.Mojsoska, BiljanaUrquhart, ErinFaleiro, Maria L.Molteni, MonicaUrra, José MiguelFalkinham, JosephMoon, YuseokVago, RiccardoFanelli, FrancescaMoore, Matthew D.Vale, Carlos Alberto Garcia DoFang, YiMoore, Robert J.Vale, CarmenFarrell, HazelMorales-Narváez, EdenVale, PauloFasullo, MichaelMoran, YehuValério, ElisabeteFathi Abdallah, MohamedMoreira, CristianaValero, ManuelFedak, GeorgeMorse, DavidVamanu, EmanuelFernandes, Ana S.Mowe, MaxineVan Der Merwe, DeonFernandes, José OliveiraMukherjee, SomnathVan Sanford, David A.Ferrara, MassimoMurakami, MakotoVance, DavidFerreras, José MiguelMurphy, CarolineVandooren, JenniferFink-Gremmels, JohannaMuruganandam, GopinathVarga, ElisabethFinley, NatoshaMurugesan, G. RajVasylieva, NataliaFitzgerald, DavidMutiga, Samuel K.Vaughan, Martha MFlorens, NansMutsaers, HenricusVaz Moreira, IvoneFlores-Moya, AntonioMuyldermans, SergeVenâncio, ArmandoFologea, DanielNabok, AlexeiVenkatadri, RajkumarFonfria, ElenaNagahama, MasahiroVerheecke, CarolFont, GuillerminaNagai, SatoshiVerhulst, AnjaFoster, TimNagashima, HitoshiVerma, ArjunFourtounas, CostasNagraj, VinayVetter, RichardFox, Edward MNakaminami, HidemasaVidal, TâniaFradin, ChantalNakamura, NorikoVidic, JasminaFreire, JoaoNakashima, SouichiVilcinskas, AndreasFribley, Andrew M.Nanjappa, DeepakVillani, AlessandraFrisan, TeresaNath, ArijitVillar-Briones, AlejandroFrisvad, Jens ChristianNathanail, AlexisVimercati, LuigiFrucht, David M.Natskoulis, PantelisVoss, Kenneth A.Fujinaga, YukakoNeil, CrickmoreVozzi, GiovanniFujita, MasakiNestorovich, Ekaterina M.Vudathala, DaljitFülöp, TiborNevalainen, TimoWagg, AdrianFuly, AndréNg, Kenneth Kai-SingWakino, ShuFurukawa, TomohiroNIA, YacineWalker, AndrewFusco, VincenzinaNiculita-Hirzel, HélèneWan, Lam YimGalanis, AlexisNiedziela, TomaszWang, XinwenGalaverna, GianniNielsen, Vance G.Wang, ZhijunGall, BrianNishiyama, AkihitoWangchuk, PhurpaGao, YangNoguchi, TamaoWard, MicaiahGarber, Eric A.E.Norton, RaymondWaśkiewicz, AgnieszkaGarcía-Cela, EstherNossol, ConstanzeWatanabe, RyuichiGarcía-Ortega, LucíaNOVAK, BarbaraWaters, MatthewGarner, BiancaNugent, MarcWeaver, KeithGarrosa, ManuelO’Mahony, MicheálWeaver, MarkGastaldello, StefanoObrig, TomWee, JosephineGazerani, ParisaOfford, JamesWeighardt, HeikeGehring, RonetteOgashawara, IgorWeiser, Douglas C.Genitsaris, SavvasOgihara, JunWeller, Michael G.Gerhard, RalfOlson, Michael E.Weng, AlexanderGerin, DonatoOmur-Ozbek, PinarWeng, Ching-FengGerner, ChristopherOnduka, ToshimitsuWhittington, CarlGhanim Al-Rubaye, AliOno, HisayaWiedman, GregoryGhosh, SumitOppert, BrendaWiesner, JacquelineGiannantoni, AntonellaOppliger, AnneWilders, RonaldGianni, AretiOsborne, NicholasWillerth, Stephanie MGiardino, IdaOshiro, NaomasaWilliams, Andrew R.Gilabert-Oriol, RogerOsorio, CarlosWillis, AnusuyaGilbert, FlorenceOspina-Millán, Claudia A.Wilson, Brenda AnneGillet, DanielOstolaza, HelenaWilson, DavidGilli, FrancescaOtero, PazWilson, RodGlibert, Patricia M.Oyama, EtsukoWindham, GaryGoda, KeisukeOzanich, Richard M.Woolbright, Benjamin L.Goldsby, Richard A.Padula, AndrewWöstemeyer, JohannesGómez De Cedrón, MartaPajek, JernejWozniak-Knopp, GordanaGonkowski, SlawomirPalanisamy, Satheesh KumarWree, AndreasGorrell, RebeccaPanagou, EfstathiosWuster, WolfgangGoto, ShunsukePannek, JürgenXi, XinpingGoud, K YugenderPapageorgiou, MariaYabe, KimikoGregory-Eaves, IrenePapatheodorou, PanagiotisYaksh, TonyGris, DenisPapenbrock, JuttaYamaguchi, YoshihiroGromadzka, KarolinaPark, Hue JungYamamoto, KeiGruber, ChristianPark, Hyun-WooYamamoto, SuguruGuerillot, RomainPark, JongHoYang, WeishanGuerre, PhilippePark, Se ChangYang, Yi-YuanGuinamard, RomainPark, Sok CheonYano, ShozoGundogdu, OzanPaßlack, NadineYao, GuoruiGupta, Anupam DattaPatel, AmarYilmaz, NevalGutierrez, SantiagoPatel, HardikkumarYli-Mattila, TapaniGuzmán-Guillén, RemediosPaterson, Gavin K.Yong, Tai-soonHa, Tai HwanPatrauchan, Marianna A.Yoshihisa, TOMIOKAHaan, JisunPawlak, DariuszYoshinari, TomoyaHaesaert, GeertPazos, Antonio JuanYotsu-Yamashita, MariHallegraeff, Gustaaf M.Peacock, MelissaYu, Feng-YihHallen-Adams, HeatherPeeters, ElisabethYuzawa, YukioHans, GuyPeigneur, SteveZacchia, MiriamHarijan, Rajesh KPelin, MarcoZakrzewska, MalgorzataHarms, AlexanderPellett, SabineZallot, Remi G.Harris, Ted D.Pence, BrandtZałuski, DariuszHarrison, Lisa MPeraica, MajaZaric, SvetislavHartung, ThomasPereira, Ana LuisaZarrelli, ArmandoHarvey, PetaPeres, António M.Žatecka, EvaHasegawa, MakotoPerona, ElviraZelanis, AndréHaug, TorPerrier, JosetteZeleny, ReinhardHautbergue, GuillaumePerrone, GiancarloZeng, HulieHawlitschka, AlexanderPerry, Michael J.Zervou, Sevasti-KiriakiHellinger, RolandPetitpas, Christian M.Zhang, FanHellmann, NadjaPfohl-Leszkowicz, AnnieZhang, KaiHénaut, LuciePhillips, Gregory J.Zhang, SicaiHernández-León, AlejandroPiccinini, RenataZhao, BoxuanHernández-Martínez, PatriciaPickett, AndyZhao, RuimingHerring, SusanPicking, William D.Zhou, Carol L. EcaleHerzig, VolkerPietri, AmedeoZichel, RanHeuck, AlejandroPincus, SethZilberberg, NoamHiruma, KeiPinotti, LucianoZimba, PaulHo, MengfeiPitt, JohnZingali, RussolinaHook, IngridPla, DaviniaZobel-Thropp, Pamela A.Hopping, GenePlaczek, RichardZou, EnminHosomi, RyotaPlaza-Díaz, JulioZuliani, Juliana PavanHosório, HugoPletinck, Anneleen
Hruska, ZuzanaPollard, K. Michael


